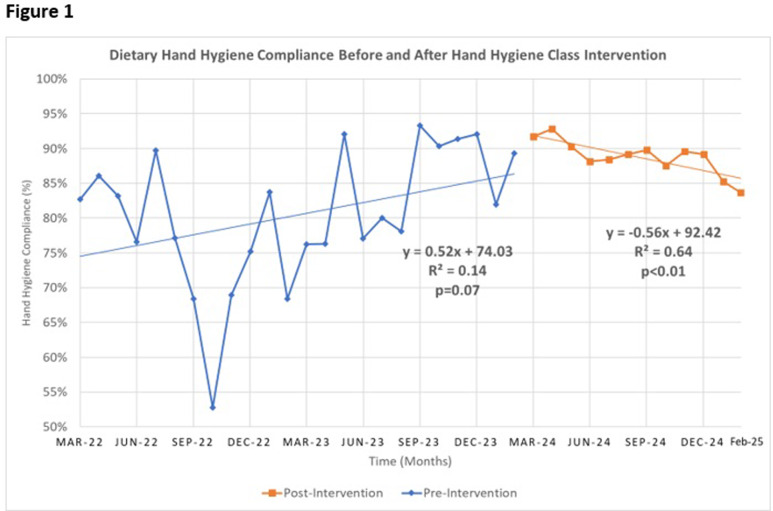# 261 Burden of Contact Isolation Associated with Methicillin Resistant Staphylococcus Aureus Colonization in a Neonatal Intensive Care Unit

**DOI:** 10.1017/ash.2026.10627

**Published:** 2026-06-23

**Authors:** Daniel Thompson, Tigre Suder, Sarah Cecil, Rachel Howard, Kimberly Blanton, Kevin Hatton, David Olafsson, Sean McTigue, Nicholas Van Sickels, Takaaki Kobayashi

**Affiliations:** 1 University of Kentucky Healthcare; 2 University of Kentucky HealthCare; 3 UK HealthCare; 4 UK Healthcare; 5 University of Kentucky

## Abstract

**Background:** Proper hand hygiene (HH) is a fundamental practice for reducing healthcare-associated infection transmission and a cornerstone of infection prevention. Dietary technicians, non-clinical food service workers who deliver patient meal trays, routinely move between patient rooms. Although many healthcare institutions have established multimodal HH compliance programs, dietary staff have historically demonstrated lower HH compliance compared with clinical personnel. HH interventions targeting this group remain understudied. We implemented an infection prevention–led HH education class for onboarding dietary staff and evaluated its association with overall dietary HH compliance. **Methods:** We conducted a quasi-experimental study at a large academic medical center. The onboarding dietary HH class began on March 4, 2024, and was delivered intermittently, averaging twice monthly, with pauses due to operational constraints, through February 28, 2025. Newly hired dietary technicians attended a single in-person, one-hour session during orientation, with no subsequent reinforcement. Instruction was conducted in an empty patient room and emphasized HH upon room entry and exit, appropriate use of alcohol-based hand rub versus soap and water, and correct personal protective equipment use aligned with transmission-based precautions. HH observations for all dietary staff were collected by trained auditors using standardized direct observation protocols. Baseline data were obtained from March 1, 2022, to February 29, 2024, and intervention data from March 1, 2024, to February 28, 2025. **Results:** Approximately 80 newly hired dietary technicians participated across 21 total classes. Mean HH compliance increased from 80 percent at baseline (95 percent CI, 76.4–84.6; 4,971 observations) to 89 percent during the intervention period (95% CI, 87.2–90.4; 2,932 observations; p < 0.05 by Wilcoxon rank-sum test). Compliance variability decreased, with standard deviation declining from 9.7 percent to 2.3 percent and monthly minimum compliance increasing from 53 percent to 84 percent. In segmented linear regression analysis, HH compliance demonstrated a non-significant upward trend during the pre-intervention period (p = 0.07). Following the intervention, a small but statistically significant downward trend over time was observed (p < 0.01). **Conclusions:** An onboarding dietary HH education class was associated with higher mean HH compliance and reduced variability among dietary staff. However, improvements were not durable, likely reflecting the limited reach and lack of reinforcement inherent to an intermittently delivered, onboarding-only intervention and a potential ceiling effect within an existing multimodal HH compliance program. These findings emphasize the importance of reinforcement strategies when designing HH interventions for dietary staff to sustain improvements and enhance patient safety.

**Figure:**